# Genome-wide association study of electrocardiographic and heart rate variability traits: the Framingham Heart Study

**DOI:** 10.1186/1471-2350-8-S1-S7

**Published:** 2007-09-19

**Authors:** Christopher Newton-Cheh, Chao-Yu Guo, Thomas J Wang, Christopher J O'Donnell, Daniel Levy, Martin G Larson

**Affiliations:** 1 The National Heart, Lung, and Blood Institute's Framingham Heart Study, Framingham, MA, USA; 2 Program in Medical and Population Genetics, Broad Institute of Massachusetts Institute of Technology and Harvard University, Cambridge, MA, USA; 3 Cardiology Division, Massachusetts General Hospital, Harvard Medical School, Boston, MA, USA; 4 Departments of Mathematics and Statistics, Boston University, Boston, MA, USA; 5 National Heart, Lung and Blood Institute, Bethesda, MD, USA; 6 On behalf of the Framingham Heart Study 100K Cardiovascular Disease Working Group

## Abstract

**Background:**

Heritable electrocardiographic (ECG) and heart rate variability (HRV) measures, reflecting pacemaking, conduction, repolarization and autonomic function in the heart have been associated with risks for cardiac arrhythmias. Whereas several rare monogenic conditions with extreme phenotypes have been noted, few common genetic factors contributing to interindividual variability in ECG and HRV measures have been identified. We report the results of a community-based genomewide association study of six ECG and HRV intermediate traits.

**Methods:**

Genotyping using Affymetrix 100K GeneChip was conducted on 1345 related Framingham Heart Study Original and Offspring cohort participants. We analyzed 1175 Original and Offspring participants with ECG data (mean age 52 years, 52% women) and 548 Offspring participants with HRV data (mean age 48 years, 51% women), in relation to 70,987 SNPs with minor allele frequency ≥ 0.10, call rate ≥ 80%, Hardy-Weinberg p-value ≥ 0.001. We used generalized estimating equations to test association of SNP alleles with multivariable-adjusted residuals for QT, RR, and PR intervals, the ratio of low frequency to high frequency power (LF/HFP), total power (TP) and the standard deviation of normal RR intervals (SDNN).

**Results:**

Associations at p < 10^-3 ^were found for 117 (QT), 105 (RR), 111 (PR), 102 (LF/HF), 121 (TP), and 102 (SDNN) SNPs. Several common variants in *NOS1AP *(4 SNPs with p-values < 10^-3^; lowest p-value, rs6683968, p = 1 × 10^-4^) were associated with adjusted QT residuals, consistent with our previously reported finding for *NOS1AP *in an unrelated sample of FHS Offspring and other cohorts. All results are publicly available at NCBI's dbGaP at http://www.ncbi.nlm.nih.gov/projects/gap/cgi-bin/study.cgi?id=phs000007.

**Conclusion:**

In the community-based Framingham Heart Study none of the ECG and HRV results individually attained genomewide significance. However, the presence of *bona fide *QT-associated SNPs among the top 117 results for QT duration supports the importance of efforts to validate top results from the reported scans. Finding genetic variants associated with ECG and HRV quantitative traits may identify novel genes and pathways implicated in arrhythmogenesis and allow for improved recognition of individuals at high risk for arrhythmias in the general population.

## Background

Quantitative non-invasive measures of cardiac electrical activity recorded in electrocardiographic (ECG) and heart rate variability (HRV) studies are widely available in community-based samples and have been found to be predictive of cardiovascular events including sudden cardiac death [1-7]. Aggregation of these measures within families suggests a heritable component.

Prior studies, including our own, have reported the heritability of common indices of myocardial repolarization and HRV measures. The heritability of electrocardiographic QT interval duration, a measure of myocardial repolarization, has been reported to be approximately 35%, indicating that 35% of the variability in adjusted QT interval duration is attributable to heritable factors [8-11]. Electrocardiographic RR interval, or its inverse heart rate, has been observed to have a heritability ranging from 32–40% in family studies [12,13] and 54–77% in twin studies [11,14,15]. Electrocardiographic PR interval has reported heritability estimated at 34% [15]. We have previously reported the heritability of HRV measures including the ratio of low frequency to high frequency power, total power and the standard deviation of normal RR intervals [16].

The heritability of ECG and HRV traits suggests there is a significant genetic component to the determination of these measures of myocardial repolarization, sinus node function, atrioventricular conduction, and autonomic function. Rare variants in ion channel genes have been implicated in rare Mendelian Long QT Syndromes. Historically, efforts to identify genetic determinants of common, complex traits have been focused on linkage or association of a small number of such biologic candidate genes with limited success and conflicting results.

Genome-wide association studies (GWAS) offer the opportunity to test a large fraction of common genetic variation using high-throughput genotyping arrays. Such studies have recently identified genes previously unrecognized to contribute to disease, including complement factor H and age-related macular degeneration [17], *INSIG2 *and obesity [18], and the *IL23R *and inflammatory bowel disease [19]. To date the most convincing association with an ECG or HRV trait is that of a common variant in *NOS1AP *with QT interval variation identified through GWAS, reported by us in collaboration with others [20]. This study, using a fixed genotyping array to survey 100,000 variants, discovered a novel gene involved in myocardial repolarization and demonstrated the power of such unbiased methods to identify previously unrecognized genes or pathways involved in cardiovascular physiology. One such array, the Affymetrix 100K GeneChip has been shown to capture about 30% of common genetic variants among the European ancestry HapMap CEU sample [21]. We therefore sought to relate 70,987 common genetic variants genotyped on this array to ECG and HRV phenotypes in participants in the Framingham Heart Study as a first step toward identifying genetic variants that influence important cardiovascular traits.

## Methods

### Study sample

The Framingham Heart Study is a cohort of predominantly European ancestry; the study sample is more fully described in the Overview Methods section [22]. ECG traits were measured between 1968 and 1975 using the entire sample of FHS Original and Offspring Cohort participants (examination cycles 11 and 1, respectively) with available measures, and free of prevalent coronary heart disease, atrial fibrillation and anti-arrhythmic medication use (n = 7356). The heritability sample for ECG analyses comprised 1951 individuals in 355 pedigrees. HRV traits were measured between 1983 and 1987 using the entire sample of FHS Original (examination cycle 18) and Offspring Cohort participants (examination cycle 3) with available measures, and free of prevalent myocardial infarction, congestive heart failure, atrial fibrillation, diabetes, and antihypertensive or cardioactive medication use (n = 1966). The heritability sample for HRV traits comprised 747 subjects in 307 pedigrees. The sample examined for GWAS included the subset of individuals on the FHS Related plates (n = 1175 for ECG analyses, n = 548 for HRV analyses). Each study participant provided written informed consent for genetic analyses and the study was approved by the Boston University Medical Center Institutional Review Board.

### Phenotype definition

Digital caliper measurements were made on scanned paper ECGs recorded at 25 mm/sec, with good reproducibility as previously shown [9]. QT interval duration on the ECG was taken from the onset of the QRS to the end of the T wave or the nadir between the T wave and U wave if present, as previously described [9]. The QT phenotype was defined as the averaged, standardized residuals from sex-, lead-(II, V_2_, V_5_) and cohort-specific linear regression on age and RR interval. RR interval duration was measured as the time in msec from one R wave to the next R wave. The RR phenotype was defined as the averaged, standardized residuals from sex-, lead-(II, V_2_, V_5_), and cohort-specific linear regression on age. PR interval duration was measured from the onset of the P wave to the onset of the QRS interval on lead II only. The PR phenotype was defined as the standardized residual from sex- and cohort-specific linear regression on age and RR interval.

Heart rate variability measures were extracted from two hour ambulatory ECG recordings as previously described [16,23]. We excluded recordings with nonsinus rhythm, >10% premature beats, <1 hour recording time, or processed time <50% recording time. Fast Fourier transform analysis was performed on 100-second blocks of RR interval data from 32 Hz recordings (Cardiodata Corp) sampled at 180 samples/second (Mortara Instrument Co). Power density spectra were averaged across all 100-second blocks. HRV phenotypes include two frequency domain measures, ratio of low frequency to high frequency power (LF/HF) and total power (TP) and one time domain measure: standard deviation of normal RR intervals (SDNN). The phenotype studied was the standardized, log-transformed residuals from sex and cohort-specific linear regression of HRV trait on age, heart rate, systolic and diastolic blood pressures, coffee intake and alcohol intake [23].

### Genotyping and annotation

Affymetrix 100K SNP genotyping is described in the Overview Methods section [22]. Genotype annotation using dbSNP and UCSC Genome Browser [24,25] are described in the Overview Methods section [22].

### Statistical analysis

The general statistical methods for linkage and GWA analyses are described in the Overview Methods section [22]. Heritability was estimated using the variance components methods implemented in SOLAR [26]. The primary analysis of SNP associations with the 6 ECG and HRV phenotypes involved use of linear regression of minor allele copy number (additive genetic model) on phenotype using generalized estimating equations (GEE) to account for relatedness among individuals as described in the Overview [22]. Secondary analyses involved family-based association testing using FBAT [27] and linkage using SOLAR [25] after exact identity-by-descent estimation using Merlin [28] on a subset of 11,200 SNPs and STRs. Affymetrix 100K GeneChip SNPs tested for association with phenotypes included the 70,987 autosomal SNPs with minor allele frequency ≥ 10%, call rate ≥ 80% and Hardy-Weinberg equilibrium p-value ≥ 0.001. For all results, nominal p-values are shown, unadjusted for multiple tests. For positive control analyses of rs10494366, we accessed HapMap CEU genotypes at the *NOS1AP *locus December 11, 2005 (http://www.hapmap.org). Correlation (r^2^) among HapMap CEU SNPs was determined using HaploView 4.0 beta 11 (http://www.broad.mit.edu/mpg/haploview).

## Results

Clinical characteristics of the FHS sample of 1345 subjects are presented in the Overview [22]. Table 1 displays the variables that were studied in our analyses of ECG and HRV traits. Further information on these traits can be found at http://www.ncbi.nlm.nih.gov/projects/gap/cgi-bin/study.cgi?id=phs000007.

**Table 1:** Heritability of electrocardiographic and heart rate variability phenotypes examined in the Framingham 100K project

Phenotype	Acronym	Heritability sample	Original Exam	Offspring Exam	Adjustment*	Heritability^† ^(SE)
**Electrocardiographic phenotypes**						

QT sex-pooled	QTPOOL	1951	11	1	Age, RR, sex, cohort	0.39 (0.04)^†^
QT men only	QTMEN**	900	11	1	Age, RR, cohort	0.43 (0.08)
QT women only	QTWOMEN**	1051	11	1	Age, RR, cohort	0.46 (0.04)
RR sex-pooled	RRPOOL	1951	11	1	Age, sex, cohort	0.29 (0.04)
RR men only	RRMEN**	900	11	1	Age, cohort	0.28 (0.08)
RR women only	RRWOMEN**	1051	11	1	Age, cohort	0.31 (0.07)
PR sex-pooled	PRAdjRRPOOL	1950	11	1	Age, RR, sex, cohort	0.34 (0.04)

**Heart rate variability phenotypes**						

SDNN sex-pooled	SDNNHRV	747	18	3	Age, heart rate, SBP, DBP, coffee & alcohol intake	0.32 (0.09)^†^
Total power sex-pooled	TOTPWRHRV	747	18	3	Age, heart rate, SBP, DBP, coffee & alcohol intake	0.41 (0.10)^†^
Low frequency/high frequency power sex-pooled	LFHFHRV	747	18	3	Age, heart rate, SBP, DBP, coffee & alcohol intake	0.36 (0.10)^†^

All phenotypes for ECG and HRV traits are shown (including pre-specified secondary traits not examined for this report), including covariates adjusted for in linear regression models. Heritability estimates were generated in the superset of individuals with phenotype values in families (n ≤ 1951 for ECG traits, n ≤ 747 for HRV traits). For electrocardiographic traits there were 1175 individuals with genotypes and for heart rate variability there were 548 individuals with genotypes in the 100K analyses. All results are publicly available at http://www.ncbi.nlm.nih.gov/projects/gap/cgi-bin/study.cgi?id=phs000007.

*Separate regression models were created by cohort and sex for ECG and HRV measures. ^†^All heritability estimates <0.001.

**Prespecified secondary results in GWAS pipeline but not analyzed in this report (available at http://www.ncbi.nlm.nih.gov/projects/gap/cgi-bin/study.cgi?id=phs000007). QT and HRV heritability estimates are consistent with previous reports in largely overlapping sample [9,16].

### ECG and HRV measures are heritable traits

We have previously shown in the Framingham Heart Study that electrocardiographic QT interval duration, adjusted for RR interval, age, and sex has substantial heritability. In the current study, including a subset from the original report, heritability was estimated at 0.39 (Table 1) [9]. We have also previously shown that HRV phenotypes show familial aggregation [16]; in the current report, after adjustment for covariates, heritability estimates were 0.36 (LF/HF), 0.41 (TP), and 0.32 (SDNN) (Table 1). For the electrocardiographic RR interval (inverse heart rate), adjusted for age and sex, in the related sample of FHS original and offspring participants, we observed a heritability of 0.29. For the electrocardiographic PR interval, adjusted for age, sex and RR interval, we observed a heritability of 0.34.

### Association tests approximate the null distribution

After filtering all autosomal SNPs on call rate ≥ 80%, minor allele frequency ≥ 0.10 and Hardy-Weinberg p-value ≥ 0.001, we observed a distribution of the 70,987 p-values that approximated a null distribution. The proportions of p < 0.0001 or p < 0.001 averaged across all six ECG and HRV phenotypes were 0.00018 and 0.0016, respectively (Table 2). The proportion of tests with a p ≥ 0.10 was 0.89. The p-value distributions were stable across increasingly stringent call rate thresholds and showed only a minor trend toward fewer excess p-values with increasing minor allele frequency (data not shown).

**Table 2:** Proportion of association results at different p-value thresholds

	QT	RR	PR	LF/HF	Tot Power	SDNN	Average
**p < 0.0001**	0.000127	0.000197	0.000169	0.000281	0.000197	0.000112	0.000180
**p < 0.001**	0.00165	0.00149	0.00156	0.00143	0.00172	0.00145	0.00155
**p > 0.10**	0.891	0.886	0.882	0.895	0.895	0.893	0.890

Shown are the proportion of p-values for single SNP association tests using GEE which are more extreme than 0.0001, 0.001 and less extreme than 0.10 for the six electrocardiographic and heart rate variability traits, after excluding SNPs with call rate <80%, minor allele frequency <10%, Hardy-Weinberg equilibrium p-value < 0.001. The proportions observed closely approximate the proportions expected for a null distribution – 0.0001, 0.001 and 0.90.

### Genome-wide association results

Results can be found at http://www.ncbi.nlm.nih.gov/projects/gap/cgi-bin/study.cgi?id=phs000007. From the primary GEE analyses, the strongest associations for the QT, RR and PR phenotypes were for SNPs rs10507380 (p = 8.4 × 10^-6^), rs2179896 (p = 1.7 × 10^-5^), and rs882300 (p = 3.2 × 10^-7^), respectively (Table 3). From the primary GEE analyses, the strongest associations for LF/HF, TP, SDNN were for SNPs rs1395479 (p = 6.9 × 10^-6^), rs9315385 (p = 7.7 × 10^-6^), and rs2966762 (p = 2.0 × 10^-5^), respectively (Table 3). SNP associations by GEE analysis with nominal p < 10^-3 ^were found for 117 (QT), 105 (RR), 111 (PR), 102 (LFHFP), 121 (TP), and 102 SNPs (SDNN).

**Table 3:** Top GEE association results for electrocardiographic phenotypes

Trait	SNP rank	rs ID	Chromosome	Physical Position	GEE p-value	FBAT p-value	Gene region (within 60 kb)
**Top 8 QT interval SNPs**							

QT	1	rs10507380	13	26777526	**8.4 × 10^-6^**	0.665	*RPL21*
QT	2	rs2726920	11	108252434	**4.2 × 10^-5^**	0.865	*DDX10*
QT	3	rs763552	8	31564949	**4.7 × 10^-5^**	0.033	*NRG1*
QT	4	rs366307	5	150527337	**4.9 × 10^-5^**	0.104	*ANXA6*
QT	5	rs3858646	12	114038413	**7.7 × 10^-5^**	1.3 × 10^-4^	
QT	6	rs1558139	19	15858564	**8.2 × 10^-5^**	6.0 × 10^-5^	*CYP4F2*
QT	7	rs1695508	7	28739796	**8.5 × 10^-5^**	0.002	
QT	8	rs10495588	2	12153871	**8.7 × 10^-5^**	0.001	

**Top 8 RR interval SNPs**							

RR	1	rs2179896	14	53945264	**1.7 × 10^-5^**	0.032	
RR	2	rs10518674	15	50105438	**1.9 × 10^-5^**	0.033	*MAPK6*
RR	3	rs321967	7	77959200	**2.6 × 10^-5^**	0.018	*MAGI2*
RR	4	rs4319121	8	53668308	**3.6 × 10^-5^**	0.571	
RR	5	rs844429	10	80016392	**4.5 × 10^-5^**	0.032	
RR	6	rs4345013	22	25706391	**4.9 × 10^-5^**	0.227	
RR	7	rs2643191	3	165861395	**5.2 × 10^-5^**	2.5 × 10^-4^	
RR	8	rs2932529	1	112905406	**5.7 × 10^-5^**	0.232	*CAPZA1*

**Top 8 PR interval SNPs**							

PR	1	rs882300	2	136809987	**3.2 × 10^-7^**	0.133	
PR	2	rs10518795	15	53131579	**1.1 × 10^-5^**	0.062	
PR	3	rs7201988	16	9516007	**3.0 × 10^-5^**	0.001	
PR	4	rs2096767	11	102296609	**3.1 × 10^-5^**	0.031	*MMP13*
PR	5	rs2831936	21	28922444	**3.1 × 10^-5^**	0.011	
PR	6	rs10516736	4	82325666	**5.1 × 10^-5^**	0.014	*BMP3*
PR	7	rs4488182	11	41032137	**5.4 × 10^-5^**	0.008	
PR	8	rs10489798	1	96957067	**6.2 × 10^-5^**	0.086	*PTBP2*

**Top 8 LF/HF SNPs**							

LF/HF	1	rs1395479	4	178693340	**6.9 × 10^-6^**	0.034	*NEIL3*
LF/HF	2	rs2215456	12	40596176	**1.3 × 10^-5^**	0.002	
LF/HF	3	rs1336938	13	88369006	**1.6 × 10^-5^**	0.003	
LF/HF	4	rs796184	13	88515431	**1.9 × 10^-5^**	0.003	
LF/HF	5	rs4669749	2	11643262	**2.0 × 10^-5^**	0.113	*GREB1*
LF/HF	6	rs1871841	8	13674417	**2.1 × 10^-5^**	0.015	
LF/HF	7	rs721691	7	128485610	**3.0 × 10^-5^**	0.026	
LF/HF	8	rs10509700	10	97884521	**4.2 × 10^-5^**	0.864	

**Top 8 Total Power SNPs**							

TP	1	rs9315385	13	35561302	**7.7 × 10^-6^**	0.031	*DCAMKL1*
TP	2	rs2276886	4	77285607	**1.5 × 10^-5^**	0.004	*CXCL9*
TP	3	rs726698	2	35366992	**2.2 × 10^-5^**	0.001	
TP	4	rs1330948	13	106192232	**2.4 × 10^-5^**	0.016	
TP	5	rs2283064	7	125719004	**3.0 × 10^-5^**	0.042	*GRM8*
TP	6	rs10515199	5	74135390	**3.8 × 10^-5^**	0.113	
TP	7	rs1407709	1	184794336	**4.0 × 10^-5^**	0.042	
TP	8	rs1723482	2	47318190	**5.5 × 10^-5^**	0.040	

**Top 8 SDNN SNPs**							

SDNN	1	rs2966762	5	109411118	**2.0 × 10^-5^**	0.024	
SDNN	2	rs286751	5	107430837	**2.1 × 10^-5^**	4.1 × 10^-5^	*FBXL17*
SDNN	3	rs1013621	8	52963944	**4.0 × 10^-5^**	0.018	
SDNN	4	rs1378506	5	109419129	**4.5 × 10^-5^**	0.110	
SDNN	5	rs1866559	2	27247237	**5.3 × 10^-5^**	0.405	*CGREF1*
SDNN	6	rs2049161	18	4117583	**5.9 × 10^-5^**	0.036	
SDNN	7	rs9297393	8	108356753	**6.4 × 10^-5^**	0.587	*ANGPT1*
SDNN	8	rs7012655	8	52740967	**8.4 × 10^-5^**	0.007	

The top 8 association results for ECG phenotypes QT, RR and PR interval and for HRV phenotypes low frequency to high frequency power (LF/HF), total power (TP) and standard deviation of normal RR intervals (SDNN) are shown from a total 70,987 SNPs tested in additive genetic models using GEE. Chromosome and physical position using NCBI build 35 reference sequence (hg17) are shown. The corresponding FBAT p-value is shown (note: the number of informative families varies substantially). Genes within 60 kb of a SNP are shown.

### Positive controls in *NOS1AP *support the promise of GWAS in this sample

We have previously reported the association of a common variant rs10494366 (MAF 38%) in the *NOS1AP *gene with adjusted QT interval variation in 3 independent cohorts, including an unrelated set of Framingham Heart Study participants (on the Unrelated Plates), as part of a three-stage genome-wide association study [20]. In the current report, we have now validated the association of rs10494366, genotyped on the Affymetrix 100K GeneChip array, with QT interval duration in the Related Plate set examined (nominal 2-sided p = 0.0009, rank in 100K analysis #102, call rate 91%). Moreover, among the 117 SNPs on the array associated with QT with p < 0.001 there were three additional associated SNPs that were in or near *NOS1AP*, *all partially correlated *with rs10494366: rs6683968 (p = 0.0001, MAF 32%, 100K rank #10, r^2 ^= 0.05 to rs10494366 in HapMap CEU, call rate 93%), rs945713 (p = 0.0002, r^2 ^= 0.35, MAF 42%, rank #23, call rate 99%) and rs1932933 (p = 0.0004, r^2 ^= 0.63, MAF 39%, rank #42, call rate 99%). For illustrative purposes, we further considered a two-staged design in which one genotyped all SNPs associated with the QT phenotype with p < 0.001 (n = 117) found in the Related Plate set 100K analysis in an additional approximately 1500 independent Framingham offspring cohort participants on the unrelated plate set (the reverse of the order in which this SNP was actually genotyped). In such a design the p-value for SNP rs10494366 of 0.0009 would rise to a combined p-value of 8.9 × 10^-6 ^on joint analysis of the two samples.

### Suggestive linkage results

We observed suggestive evidence of linkage to the following phenotypes with LOD scores exceeding 2.2: LOD 2.50 on chromosome 3 (8.72 Mb) for QT interval, which has previously been reported for a largely overlapping sample using microsatellite markers [9]; 2.52 on chromosome 17 (74.21 Mb) for QT interval; 2.98 on chromosome 4 (124.75 Mb) for PR interval; 2.39 on chromosome 15 (75.51 Mb) for LF/HF, and 2.19 on chromosome 11 (4.40 Mb) for TP (Table 4).

**Table 4:** Suggestive linkage results for ECG and HRV phenotypes

phenotype	maximum LOD	SNP	chromosome	position (Mb)	1.5 LOD CI
**ECG**					

PR	2.98	rs1011725	4	124.75	113.07 – 138.58
QT	2.52	rs10512617	17	74.21	66.43 – 76.22
QT	2.50	rs164462	3	8.72	5.49 – 13.81

**HRV**					

LF/HF	2.39	rs10519165	15	75.51	67.57 – 79.33
TP	2.19	rs10500600	11	4.40	0.80 – 11.77

Shown are the linkage results exceeding a maximum multipoint LOD score of 2.0 for electrocardiographic QT, RR and PR intervals and the ratio of low frequency to high frequency power, total power and the standard deviation of normal RR intervals. The SNP at the maximum LOD score is shown as well as the confidence interval spanning the 1.5 LOD drop from the maximum.

Mb = megabase; CI = confidence interval; ECG = electrocardiogram; PR = PR interval adjusted for age, sex, RR interval; QT = QT interval adjusted for age, sex, RR interval; HRV = heart rate variability; LF/HF = ratio of low frequency to high frequency power; TP = total power.

### Candidate genes and QT, RR interval traits

Because use of a genome-wide p-value threshold may be overly conservative for candidate genes directly implicated in cardiovascular physiology, we conducted a secondary analysis of SNPs within 60 kb of candidate genes for ECG and HRV traits. Among 88 SNPs (MAF ≥ 0.10, call rate ≥ 0.8, HWE p-value ≥ 0.001) in 9 genes implicated in congenital Long QT Syndromes or QT interval duration, only SNPs in *NOS1AP *had p < 0.05 for association with QT interval duration (Table 5). Among 35 SNPs in 8 adrenergic receptor genes, SNPs with nominal p < 0.05 included two for RR interval, one for PR interval, none for LF/HF power, two for total power and 7 for SDNN (Table 5).

**Table 5:** Candidate gene associations with electrocardiographic and heart rate variability traits

Candidate Gene	SNP	Chrom	Physical position	Minor allele frequency	Phenotype	GEE P-value
**QT interval**						

*NOS1AP*	rs10494365	1	158815647	0.11	QT	0.01
*NOS1AP*	rs10494366	1	158817343	0.39	QT	9.0 × 10^-4^
*NOS1AP*	rs6683968	1	158923070	0.32	QT	1.0 × 10^-4^
*NOS1AP*	rs347311	1	159035565	0.28	QT	0.01
*NOS1AP*	rs945713	1	158867328	0.42	QT	2.4 × 10^-4^
*NOS1AP*	rs1932933	1	158849704	0.39	QT	4.3 × 10^-4^

**RR interval**						

*ADRA1A*	rs10503797	8	26659190	0.11	RR	0.04
*ADRB2*	rs10515621	5	148226737	0.14	RR	0.04

**PR interval**						

*ADRA1A*	rs520180	8	26765839	0.25	PR	0.04

**LF/HF power**						

*NA*						

**Total power**						

*ADRA1B*	rs2195926	5	159255501	0.31	TP	0.01
*ADRB2*	rs9325124	5	148229011	0.44	TP	0.03

**SDNN**						

*ADRA1A*	rs10503791	8	26611431	0.17	SDNN	0.02
*ADRA1B*	rs2195926	5	159255501	0.31	SDNN	0.03
*ADRA1A*	rs10503788	8	26611063	0.18	SDNN	0.01
*ADRA1A*	rs10503789	8	26611248	0.18	SDNN	0.01
*ADRA1A*	rs10503790	8	26611360	0.18	SDNN	0.01
*ADRA1A*	rs10503794	8	26612682	0.18	SDNN	0.01
*ADRA1A*	rs7000280	8	26611857	0.18	SDNN	0.01

Associations with QT interval of SNPs within 60 kb of candidate genes implicated in congenital Long QT Syndrome or QT duration and with RR and PR interval, low frequency to high frequency power (LF/HF), total power, and the standard deviation of normal RR intervals (SDNN) of SNPs within 60 kb of adrenergic receptor genes at a nominal p < 0.05 are shown.*

*SNPs with minor allele frequency <10%, call rate <80% or Hardy-Weinberg p-value < 0.001 were excluded. Candidate gene loci tested for association with QT interval duration include 88 SNPs within 60 kb of the following genes: *KCNQ1*, *KCNH2*, *SCN5A*, *ANK2*, *KCNE1*, *KCNE2*, *KCNJ2*, *CACNA1C *and *NOS1AP*. Candidate gene loci tested for association with RR, PR, LF/HF, total power, and SDNN include 35 SNPs within 60 kb of the following genes: *ADRB1*, *ADRB2*, *ADRA1A*, *ADRA1B*, *ADRA1D*, *ADRA2A*, *ADRA2B*, and *ADRA2C*.

## Discussion

We confirmed the heritability of electrocardiographic RR interval (inverse heart rate) and PR interval and reproduced our previously demonstrated findings that QT interval and HRV traits are heritable in our community-based European ancestry sample [9,16]. We have tested 70,987 common genetic variants (MAF ≥ 10%) for association with six heritable electrocardiographic and heart rate variability phenotypes, which have been shown to be associated with adverse cardiovascular outcomes, including sudden cardiac death. No result attained a genome-wide significance threshold, such as p < 1 × 10^-7 ^required for Bonferroni correction for six traits and 70,987 SNPs. The failure to achieve a genome-wide p-value threshold reflects the massive penalty incurred by testing so many hypotheses and the limited power to achieve such a p-value given the modest effects of common variants that contribute to complex traits. However, the presence among the test results with nominal p < 0.001 for association with QT interval duration of common variants at the *NOS1AP *locus, a recently identified and multiply replicated myocardial repolarization gene [20], supports the presence of some true positive results among the mostly false positive findings.

Strengths of our study include the moderate-sized, well-characterized community-based sample, without ascertainment on phenotype, and the precision of the ECG and HRV measurements as supported by their substantial heritability. Some limitations pertain. Our study had limited power to detect modest genetic effects in a single stage. The Affymetrix 100K GeneChip, a first generation genotyping array, has patchy coverage of common genetic variation, leaving many regions untested. Our sample is almost exclusively of European ancestry, potentially limiting extension to other populations with different linkage disequilibrium patterns or environmental exposures.

## Conclusion

Staged GWAS designs in which a modest number of results from GWAS in the first stage are tested in a second independent sample and the combined statistical evidence considered, are powerful and efficient approaches to identify common variants associated with complex traits [29,30]. Such approaches may be warranted for follow up of findings from this study. Through such approaches we hope to find additional genetic factors that could improve our understanding of human biology, serve as targets for novel therapeutics or contribute to improved identification of individuals at high risk for cardiovascular events. Our report and the web posting of all results from 70,987 SNPs tested for association with ECG and HRV phenotypes in the Framingham Heart Study represents a first step in this endeavor.

## Abbreviations

ECG = electrocardiogram; FBAT = family-based association test; GEE = generalized estimating equations; GWAS = genome wide association study; HRV = heart rate variability; LF/HF = ratio of low frequency to high frequency power; LOD = log-likelihood ratio; SNP = single nucleotide polymorphism; TP = total power

## Competing interests

The authors declare that they have no competing interests.

## Authors' contributions

Drs. Larson and Newton-Cheh had full access to all of the data in the study and take responsibility for the integrity of the data and the accuracy of the data analysis.

*Study concept and design*: Newton-Cheh, Levy, O'Donnell, Larson; *Acquisition of data*: Levy, O'Donnell, Newton-Cheh; *Analysis and interpretation of data*: Newton-Cheh, Larson, O'Donnell, Levy, Guo; *Drafting of the manuscript*: Newton-Cheh; *Critical revision of the manuscript for important intellectual content*: Larson, Levy, O'Donnell, Wang; *Statistical expertise*: Guo, Larson; *Administrative, technical, or material support*: Levy, O'Donnell; *Study supervision*: Newton-Cheh, Larson, Levy, O'Donnell.
